# Critical Depressed Brain Volume Influences the Recurrence of Chronic Subdural Hematoma after Surgical Evacuation

**DOI:** 10.1038/s41598-020-58250-w

**Published:** 2020-01-24

**Authors:** Kyoung Min Jang, Hyun Ho Choi, Hah Yong Mun, Taek Kyun Nam, Yong Sook Park, Jeong Taik Kwon

**Affiliations:** Department of Neurosurgery, Chung-Ang University Hospital, Chung-Ang University College of Medicine, Seoul, Republic of Korea

**Keywords:** Risk factors, Brain injuries

## Abstract

Recurrence of chronic subdural hematoma (CSDH) frequently occurs after surgical evacuation. However, the value of follow-up postoperative imaging and measuring volumetric factors to predict recurrence are still controversial. Herein, we aimed to assess the optimal timing for follow-up referential imaging and the critical depressed brain volume for CSDH recurrence. A total of 291 patients with CSDH who underwent burr hole craniotomy between January 2012 and December 2018 were consecutively enrolled in this study. Patients’ medical records and radiologic data were evaluated to predict the recurrence and analyzed using receiver operating characteristics (ROC) and binary logistic regression. Of the 291 patients, 29 (10.0%) showed recurrence after surgical evacuation. Based on ROC analysis, comparisons of depressed brain volume pre-operation, 24 h post-operation, and 7 days post-operation showed that the depressed brain volume at 7 days after surgery featured the largest area under the curve (AUC: 0.768, 95% CI, 0.709–0.811). The cut-off value of the depressed brain volume on postoperative day 7 was 51.6 cm^3^; this value predicted the recurrence of CSDH with a sensitivity and specificity of 79.3% and 67.9%, respectively. In the multivariate analysis, the depressed brain volume (>50 cm^3^) at 7 days was the sole significant risk factor related to the recurrence of CSDH in this series (OR: 6.765, 95% CI, 2.551–17.942, p < 0.001). The depressed brain volume > 50 cm^3^ visualized on CT scans at postoperative 7 day is the critical volume affecting recurrence of CSDHs. This result could be helpful carrying in patients with CSDH to determine the proper postoperative treatment strategy.

## Introduction

Chronic subdural hematoma (CSDH) is a common condition encountered by neurosurgeons. The annual incidence of CSDH in the general population ranges from 8.2 to 17.6/100,000, and this range increases markedly with older populations^1,2^. Therefore, the proper management of CSDH is becoming increasingly important. CSDH is currently primarily managed by surgical evacuation with a burr hole craniotomy; however, recurrence after surgical evacuation is frequent, thereby requiring repeated surgical evacuations^3^.

Several factors such as brain atrophy, under-expansion, and preoperative and postoperative hematoma volumes have been evaluated as risk factors for CSDH recurrence after surgical evacuation^4–8^. CSDH frequently shows mixed densities, including hygroma and different stages of hematomas on preoperative images. Furthermore, pneumocephali also occupy subdural spaces with residual hematoma post-operation; this restricts the expansion of the brain. These factors depress the brain cortex and are possible causes for recurrence. Therefore, we hypothesized that depressed brain volumes visualized on preoperative and postoperative computed tomography (CT) were likely associated with CSDH recurrence. Although there have been many previous studies on hematoma volumes or under-expansion in the post-operative brain, the optimal timing for follow-up referential image scans and the critical depressed brain volume have not been satisfactorily evaluated. Therefore, we conducted a retrospective study to determine the risk factors for CSDH recurrence after surgical evacuation by focusing on the optimal timing to obtain follow-up referential images and the critical depressed brain volume.

## Materials and Methods

### Study population

Between January 2012 and December 2018, 297 patients with CSDH were treated with surgical evacuation in our institution. Among them, five patients were excluded owing to lack of follow-up imaging and one patient with acute intracranial hemorrhage at postoperative three days was also excluded. Finally, 291 patients were consecutively enrolled for this study. Medical variables were retrospectively reviewed for patient-related factors, including sex, age, body weight, body mass index, smoking, alcohol, hypertension, diabetes mellitus, hyperlipidemia, heart disease, liver disease, renal disease, malignancy, craniotomy history, antiplatelet medication, anticoagulant medication, platelet count, international normalized ratio (INR), and activated partial thromboplastin time (aPTT). Data on hematoma features and treatment options including bilateral hematomas, maximal midline shifting, maximal hematoma thickness, mixed density hematomas, double burr hole trephination, bilateral surgical evacuation, and depressed brain volume were also retrieved. In addition, clinical follow-up data including hospital day, required interval for complete hematoma resolution, the duration from the first burr-hole craniostomy to re-operation, and the number of required additional CT scans after seven postoperative days were collected.

This study was conducted according to the principles outlined by the Declaration of Helsinki and was approved by Chung-Ang University Hospital Institutional Review Board. The review board approved the need for informed consent was waived given the retrospective nature of the study.

### Surgical procedure

Of the 291 surgical procedures, 77 and 214 procedures were performed under general and local anesthesia, respectively. All patients underwent preoperative CT or magnetic resonance imaging (MRI) to confirm CSDH. Surgical treatment was indicated for patients with neurologically symptomatic CSDH or medically intractable headaches. Therapeutic alterations were considered through a multidisciplinary decision-making process, and informed consent was obtained from patients and their family members. The standard surgical treatment for CSDH was either single or double burr hole craniotomies and saline irrigation. A closed drainage system was inserted in 285 patients and was usually removed within three postoperative days. Antiplatelet and anticoagulant medications were routinely discontinued before the surgery, and patients on warfarin were administered intravenous vitamin K to correct INR prolongation (≥1.5). Antiplatelet and anticoagulant medications were considered for represcription depending on the patient’s condition.

### Preoperative and follow-up radiological imaging

**Figure 1:**
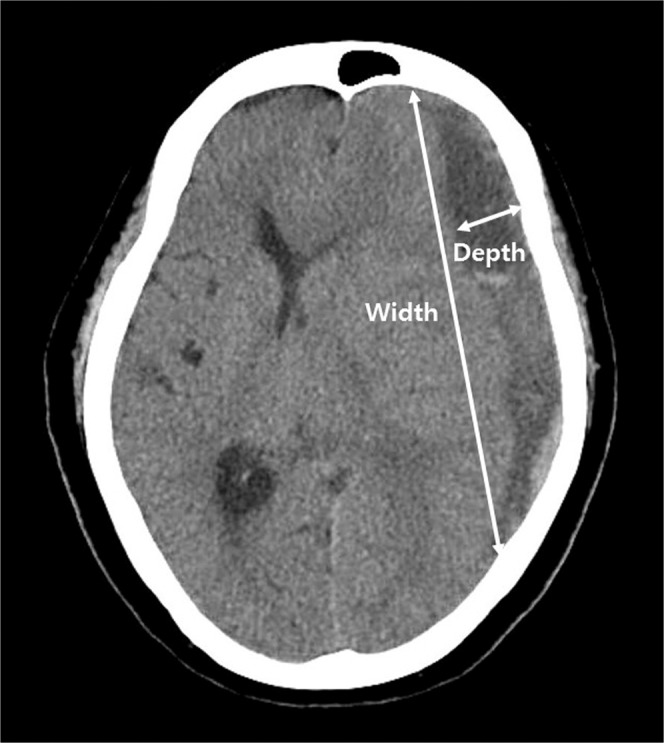
Computed tomography (CT) scan demonstrating measurements of width and depth for subdural hematoma.

The depth, width, and height dimensions of a depressed brain were measured using CT or MRI^9,10^. The maximal depth was measured as the largest diameter between the brain’s surface and the skull’s inner table in the slice near the center of the hematoma. The width was measured as the linear distance between each corner where the brain’s surface met the skull’s inner table. The height was assessed by multiplying the number of slices where hematoma was detected with the slice thickness marked on the CT scan (Fig. 1).

Depressed brain volume was calculated using the following formula:

$${\rm{Depressed}}\,{\rm{brain}}\,{\rm{volume}}\,({{\rm{cm}}}^{3})=\frac{{\rm{Depth}}\,(\text{cm})\times {\rm{Width}}\,({\rm{cm}})\times {\rm{Height}}\,\,({\rm{cm}})}{2}$$

Follow-up radiological examinations were routinely conducted within the first 24 h and at postoperative 7 days after surgery using CT. Additional CT scans was selectively conducted for patients with a large amount of residual hematoma or neurological deterioration. Recurrence was defined as increased hematoma volume in the ipsilateral subdural space and a reoperation within 2 months after the initial surgical treatment. Repeated surgical treatment was recommended for patients with medically intractable headaches or neurological deficits.

### Statistical analysis

Categorical data were expressed as frequencies and percentages, while continuous variables were presented as the mean ± standard deviation (range). The accuracy of the depressed brain volumes for predicting recurrence after surgical evacuation was determined by the receiver operating characteristic (ROC) curve. The cut-off value was defined as the highest sum of sensitivity and specificity calculated based on the Youden index. A binary logistic regression model was used for the univariate analysis of risk factors for recurrence. Multivariate analysis was performed to ensure that variables with a p-value < 0.20 in the univariate analysis were independently predictive of recurrence. The Kaplan–Meier survival curves were plotted and the log-rank test was conducted to compare time to recurrence. A two-tailed p-value < 0.05 was considered statistically significant. All data were analyzed using the MedCalc (MedCalc Software, version 19.5, Ostend, Belgium) and the Statistical Package for the Social Sciences (SPSS, Version 25, IBM, Armonk, New York).

## Results

### Baseline patient characteristics

A total of 291 patients (83 female, 28.5%; mean age: 71.8 ± 11.8 years; range: 9–95 years) were enrolled in this study. Overall, 18 (6.2%) patients had experienced craniotomies. Of all patients, 77 (26.5%) and 16 (5.5%) were prescribed antiplatelet medication and an anticoagulant agent, respectively. Bilateral hematomas were observed in 116 (39.9%) patients, and 94 (32.3%) underwent bilateral surgical evacuation. Double burr hole trephinations were performed in 70 (24.1%) patients. The maximal midline shift was 7.2 ± 4.8 mm (median: 6.5 mm, range: 1.1–21.2 mm) and maximal hematoma thickness was 19.6 ± 6.3 mm (median: 19.6 mm, range: 6.9–43.5 mm). Recurrence after surgical evacuation was detected in 29 (10.0%) patients during the follow-up. Of these patients, 27 underwent reoperation with burr hole surgery and two patients were treated with craniotomies. Baseline characteristics of patients with chronic subdural hematoma were detailed in Table 1.

**Table 1:** Baseline characteristics of patients with chronic subdural hematoma.

Total number	291
Mean age (years)	71.8 ± 11.8
Female	83 (28.5%)
Body weight	61.6 ± 11.3
Body mass index	22.9 ± 3.6
Smoking	111 (38.1%)
Alcohol	125 (43.0%)
Hypertension	160 (55.0%)
Diabetes mellitus	85 (29.2%)
Hyperlipidemia	55 (18.9%)
Heart disease	48 (16.5%)
Liver disease	30 (10.3%)
Renal disease	27 (9.3%)
Malignancy	31 (10.7%)
Craniotomy history	18 (6.2%)
Antiplatelet medication	77 (26.5%)
Anticoagulant medication	16 (5.5%)
Platelet count (×10^3^/μL)	233.4 ± 87.2
INR	1.1 ± 0.2
aPTT (sec)	30.7 ± 6.6
Bilateral hematoma	116 (39.9%)
Maximal midline shifting (mm)	7.2 ± 4.8
Maximal hematoma thickness (mm)	19.6 ± 6.3
Mixed density hematoma	141 (48.5%)
Double burr hole trephination	70 (24.1%)
Bilateral surgical evacuation	94 (32.3%)
Recurrence	29 (10.0%)

INR, International normalized ratio; aPTT, activated partial thromboplastin time.

### Comparison of depressed brain volumes

**Figure 2:**
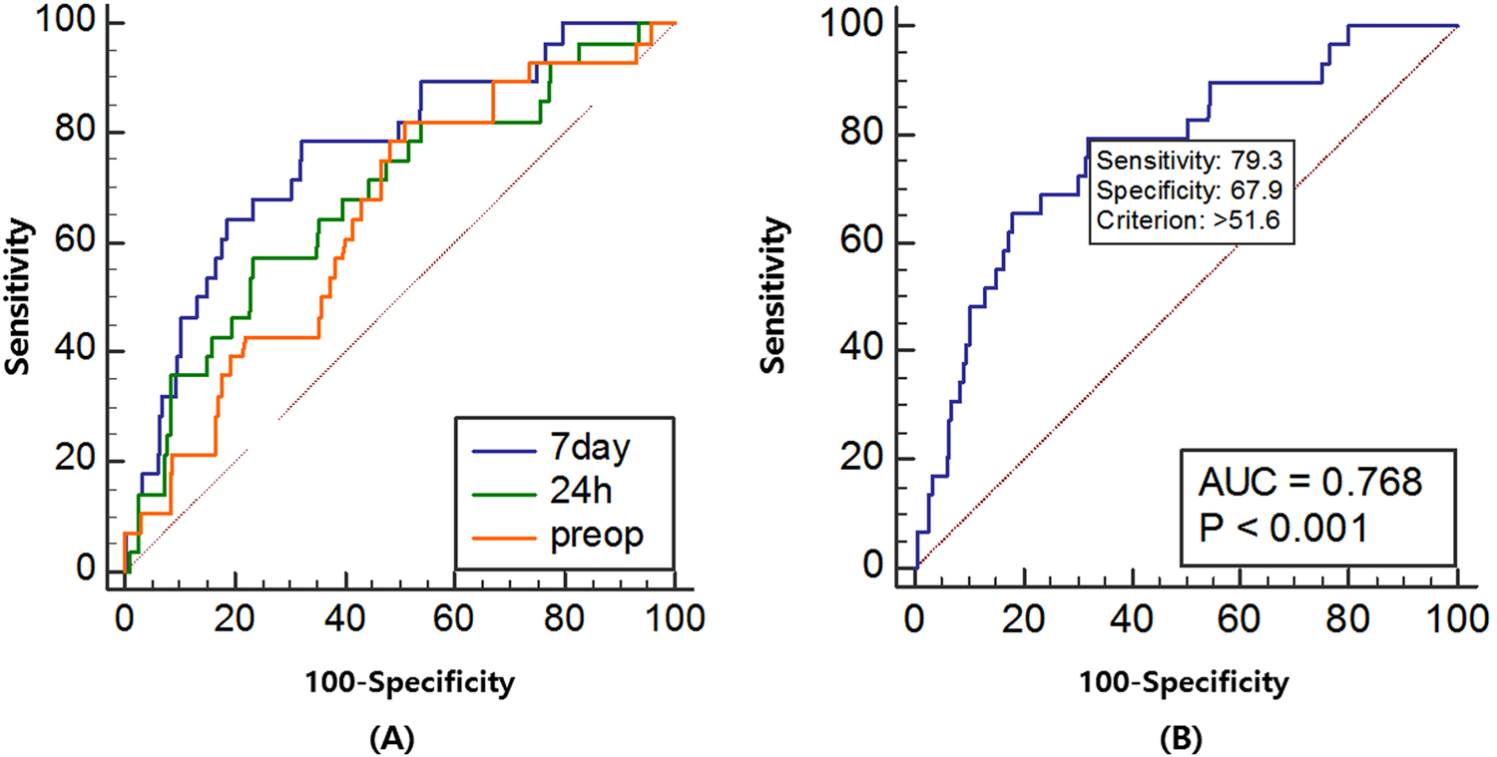
Receiver operating characteristic curve analysis for recurrence after surgical evacuation of chronic subdural hematoma. (**A**) Comparison of depressed brain volume at pre-operation, 24 h, and 7 days after surgery. (**B**) Cut-off value of depressed brain volume in CT scan at postoperative 7 days.

The depressed brain volumes were as follows: 96.5 ± 44.5 cm^3^ (median: 93.1 cm^3^, range: 13.2–245.3 cm^3^) on preoperative brain scans; 56.5 ± 36.7 cm^3^ (median: 52.8 cm^3^, range: 0–218.7 cm^3^) within 24 h; and 45.0 ± 30.4 cm^3^ (median: 41.9 cm^3^, range: 0–162.6 cm^3^) at postoperative 7 days. The ROC curve analyses that compared the predictive abilities among the depressed brain volumes showed that the AUCs of depressed brain volume were 0.643 (95% CI, 0.585–0.698) on preoperative brain scans; 0.681 (95% CI, 0.624–0.735) on scans within 24 h; and 0.768 (95% CI, 0.709–0.811) on scans at postoperative 7 days (Fig. 2A). In pairwise comparisons of ROC curves, the difference between areas was 0.038 (preoperative scan vs. 24 h scan, 95% CI, −0.070 to 0.146, p = 0.486); 0.120 (preoperative scan vs. postoperative 7 days scan, 95% CI, 0.035–0.205, p = 0.006); and 0.081 (24 h scan vs. postoperative 7 days scan, 95% CI, −0.015 to 0.178, p = 0.098) (Table 2). The depressed brain volume at postoperative 7 days showed the largest AUC among the three scans; moreover, the cut-off value of the depressed brain volume at postoperative 7 days was 51.6 cm^3^ (sensitivity: 79.3, specificity: 67.9, p < 0.001) (Fig. 2B).

**Table 2:** In paired comparison of the areas under the curve (AUC) of depressed brain volumes according to the image scan timing.

Group comparison	AUC difference	95% CI	p-value
Preoperation scan vs. 24 h scan	0.038	−0.070–0.146	p = 0.486
Preoperation scan vs. 7 day scan	0.120	0.035–0.205	**p** = **0**.**006**
24 h scan vs. 7 day scan	0.081	−0.015–0.178	p = 0.098

CI, confidence interval.

### Risk factors analysis for recurrence of CSDH

The risk factors for recurrence of CSDH were evaluated with the following variables at postoperative 7 days: sex, age, >75 years; body weight, >60 kg; body mass index, >25; smoking; alcohol; hypertension; diabetes mellitus; hyperlipidemia; heart disease; liver disease; renal disease; malignancy; craniotomy history; antiplatelet medication; anticoagulant medication; platelet count (<140 × 10^3^/μL); INR, >1.2; aPTT, >40 s; bilateral hematoma; maximal midline shifting, >5 mm; maximal hematoma thickness, >20 mm in the preoperative scan; mixed density hematoma; double burr hole trephination; bilateral surgical evacuation; and depressed brain volume (>50 cm^3^) at postoperative 7 days.

In univariate analysis, female sex, smoking, and depressed brain volume (>50 cm^3^) at postoperative 7 days were significantly associated with the recurrence of CSDH. Multivariate logistic regression analysis showed that the depressed brain volume (>50 cm^3^) at postoperative 7 days was the sole significant risk factor for CSDH recurrence (OR: 6.765, 95% CI, 2.551–17.942, p < 0.001) (Table 3).

**Table 3:** Risk factor analysis for recurrence after surgical evacuation of chronic subdural hematoma.

	Recurrence (%)		Univariate analysis	Multivariate analysis	
Variable	No (n = 262)	Yes (n = 29)	P-value	P-value	OR (95% CI)
Female	81 (30.9%)	2 (6.9%)	**0**.**016**	0.093	0.246 (0.048–1.262)
Age (years) >75	114 (43.5%)	8 (27.6%)	0.105	0.235	0.547 (0.202–1.481)
Body weight >60 kg	127 (48.5%)	14 (48.3%)	0.984		
Body mass index >25	68 (26.0%)	6 (20.7%)	0.638		
Smoking	94 (35.9%)	17 (58.6%)	**0**.**020**	0.237	1.827 (0.673–4.959)
Alcohol	109 (41.6%)	16 (55.2%)	0.165	0.789	0.870 (0.312–2.421)
Hypertension	145 (55.3%)	15 (51.7%)	0.710		
Diabetes mellitus	75 (28.6%)	10 (34.5%)	0.511		
Hyperlipidemia	50 (19.1%)	5 (17.2%)	0.810		
Heart disease	46 (17.6%)	2 (6.9%)	0.159	0.499	0.578 (0.118–2.830)
Liver disease	24 (9.2%)	6 (20.7%)	0.060	0.374	1.722 (0.520–5.706)
Renal disease	24 (9.2%)	3 (10.3%)	0.835		
Malignancy	27 (10.3%)	4 (13.8%)	0.565		
Craniotomy history	14 (5.3%)	4 (13.8%)	0.085	0.051	4.083 (0.991–16.815)
Antiplatelet medication	70 (26.7%)	7 (24.1%)	0.765		
Anticoagulant medication	15 (5.7%)	1 (3.4%)	0.614		
Platelet count (<140 × 10^3^/μL)	26 (9.9%)	4 (13.8%)	0.518		
INR > 1.2	34 (13.0%)	7 (24.1%)	0.108	0.441	1.547 (0.510–4.688)
aPTT > 40 sec	21 (8.0%)	3 (10.3%)	0.666		
Bilateral hematoma	103 (39.3%)	13 (44.8%)	0.566		
Maximal midline shifting (>5 mm)	145 (55.3%)	17 (58.6%)	0.663		
Maximal hematoma thickness (>20 mm)	117 (44.7%)	13 (44.8%)	0.927		
Mixed density hematoma	126 (48.1%)	15 (51.7%)	0.648		
Double burr hole trephination	61 (23.3%)	9 (31.0%)	0.356		
Bilateral surgical evacuation	83 (31.7%)	11 (37.9%)	0.496		
Depressed brain volume (>50 cm^3^) at postoperative 7 days	92 (35.1%)	23 (79.3%)	**<0**.**001**	**<0**.**001**	6.765 (2.551–17.942)

INR, International normalized ratio; aPTT, activated partial thromboplastin time.

**Figure 3:**
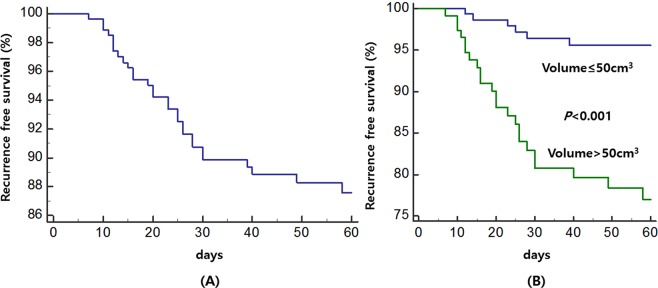
Kaplan–Meier estimates of the recurrence-free proportion in the entire patient cohort (**A**), and depressed brain volume (>50 cm^3^) at postoperative 7 days (**B**).

The Kaplan–Meier estimates of cumulative survival without recurrence are shown in Fig. 3. The overall survival rate of patients without recurrence at the 60-day follow-up was 87.6%, but the 60-day estimates differed from depressed brain volume at postoperative 7 days (≤50 cm^3^, 95.6%; >50 cm^3^, 77.0%). Cumulative survival rates without recurrence were significant for depressed brain volumes >50 cm^3^ at postoperative 7 days (p < 0.001) as per the log-rank test.

### Follow-up outcomes

Of the 291 patients, those with a cut-off volume of greater than and less than 50 cm^3^ were 108 (37.1%) and 183 (62.9%), respectively. The required time between the initial CSDH development and complete resolution of the hematoma was 87.0 (50.0–143.0) days (median [IQR]), and the interval to complete resolution was significantly longer in patients with volume greater than 50 cm^3^ than those with lesser volume (days, median [IQR]: 111.0 [67.5–161.0] vs. 69.0 [44.0–110.0], respectively, p < 0.001). Of note, more additional CT scans were required in the group of patients with volume greater than 50 cm^3^ after seven postoperative days until complete hematoma resolution, than the group with less volume (4.0 [3.0–6.0] vs. 2.0 [1.0–2.0], p < 0.001). In 29 patients with recurrence, the duration between the first burr-hole surgery and recurrence was 20.0 [12.0–28.0] days (median [IQR]). Further, CSDH recurrence was observed in 24 (22.2%) patients in the volume greater than 50 cm^3^ group and in 5 patients (2.7%) in the volume less than 50 cm^3^ group (p < 0.001).

## Discussion

Surgical evacuations with burr hole craniotomies are a well-established and effective treatment modality for CSDH; however, recurrence after surgical evacuation continues to be the main concern, as it often requires additional treatment. Therefore, it is important to confirm recurrence through proper follow-up imaging after surgery. Nevertheless, there is no consensus yet on an optimal follow-up imaging protocol, although several efforts have been made to ensure the surveillance of recurrence^11–14^. The decision to conduct follow-up postoperative CT scans can be based on the clinical status rather than just routine scanning^11–13,15^. A multicenter, retrospective analysis of 391 patients performed by Hulsbergen *et al*. revealed limited benefits of daily and routine scanning in asymptomatic patients who underwent surgical treatment for CSDH^11^. Ng *et al*. also analyzed 86 routine postoperative brain CT scans for CSDH and indicated that routine repeated daily scanning might be unnecessary regarding the overall clinical outcome^16^. In contrast, serial and repeated CT scanning within 24–48 h has been reported to show relatively high sensitivity in detecting the aggravation of traumatic intracranial hematoma and reducing potential neurological deterioration^17^. Routine scanning might allow for early diagnosis before clinical changes occur, ensuring adequate treatment strategies in the early stages and minimizing neurological sequalae in patients with recurrent chronic subdural hematomas^14^. According to our institution’s protocol, we confirmed CSDH on preoperative images and performed follow-up CT scans within 24 h and at postoperative 7 days after surgical evacuation. Comparing the ROC curves of the depressed brain volumes among three evaluations, 7 days after surgical evacuation showed the largest AUC for predicting the recurrence of CSDH, and postoperative 7 days was identified as a reasonable time for the prediction of recurrence. From the clinical relevance perspectives, the proportion of CSDH recurrence was significantly increased in patients with a depressed brain volume of greater than 50 cm^3^ as seen on the seventh postoperative day CT; moreover, the time required for complete resolution was longer. Although we could not fully guarantee the complete resolution of patients with volume less than 50 cm^3^ within that time frame, the number of additionally required CT scans was significantly lower than in those with a larger volume. Accordingly, we concluded that the optimal timing for referential imaging is postoperative 7 days rather than immediately. Additional scans should still be performed in selected patients with significant symptoms or in cases where focal signs are found during neurological examinations.

The preoperative hematoma volume and cerebral under-expansion following hematoma evacuation have been suggested to be correlated with the recurrence of CSDH; these factors are recognized to potentially create space for recurrence of a hematoma^5–7^. In Stanisic *et al*.’s study, the preoperative CSDH volume was a predictor for recurrence therefore requiring reoperation after surgical evacuation^8^. The presence of air, cerebral atrophy, and increased cerebral surface elasticity have also been reported as representative factors for under-expansion, as these factors permit persistent subdural cavity^3,18,19^. It was estimated that the collected air restricts the brain’s expansion and causes impaired obliteration of the persistent cavity, which is associated with recurrence^20^. Pre-existing cerebral atrophy is associated with recurrence, because it causes under-expansion given the excessive fibroblastic processes on the surface, leading to a persistent cavity in the subdural space^4,21^. Fukuhara *et al*.^18^ reported that the continuous mechanical compression of the cerebral surface lead to increased elastance of the surface, causing a persistent cavity in patients who had undergone hematoma evacuation.

From the pathophysiological perspective, the relationship between depressed brain volume and hematoma recurrence can be explained by repeated microbleeds and blood influxes into the persistent subdural cavity. Minor traumatic events induce the separation of the dural border cell layer, which causes damage to the bridging veins and triggers a cascade of inflammatory processes necessary for repair, including cell proliferation, granulation, and angiogenesis^22^. The outer membrane of the CSDH is derived from this cell layer. Cerebrospinal fluid and blood contain cytokines such as interleukin-6 and vascular endothelial growth factors that increase vascular permeability, thereby causing microbleeds^23,24^. Thus, the membrane has an abundance of immature and vulnerable capillary networks that induce extravasation with repeated bleeding into the hematoma cavity^25^. In addition, feeding meningeal arteries and injured bridging veins are recognized to facilitate the accumulation and growth of hematomas^26,27^. Ban *et al*. presented 72 patients with recurrent CSDH who underwent middle meningeal artery embolization. The authors indicated that the obliteration of influxes to the membrane from the dural artery prevented the accumulation of hematomas^26^. Therefore, the postoperative persistent cavity owing to depressed brain volume contributes to the recurrence of CSDH by inhibiting hemostasis with proper mechanical compression of the gap junction between the membrane and dura, and hence permitting repeated microbleeds and blood influxes. Consistent with these principles, the current study also revealed that depressed brain volume is associated with recurrence after hematoma evacuation, especially in the cases where the hematomas exceed 50 cm^3^.

Several studies have quantitatively analyzed the relation between hematoma volume or width and recurrence of CSDH^4,6,7,20,21^. The drainage volume of hematomas have been proposed as factors for recurrence^6,28^. However, the drainage volume included irrigation saline or cerebrospinal fluid, which could cause bias in the prediction of recurrence. Furthermore, hematomas exist in the three-dimensional plane, but width is a one-dimensional variable; therefore, width cannot reflect the exact volume of a hematoma. To overcome these limitations, some studies directly measured the volume. Kung *et al*.^29^ performed a quantitative analysis of volume with a computerized image analysis software and assessed the brain re-expansion rate. Stanisic *et al*.^30^ also directly measured volume using a specific software tool for 107 enrolled patients who underwent surgery for CSDH; they conducted quantitative analysis for volume excluding collected air in the subdural space. We believe that our study and analyses would be useful to neurosurgeons as they can assist in the determination of several aspects of treatment with respect to patients with CSDH. First, we adopted an integrated volume metric i.e., depressed brain volume as the variable including collected air and hematomas, which is difficult to measure each volume separately. Furthermore, while implementing the direct measurement technique, the providers can simply apply the method to clinical practice by adopting the calculated formula for CT scans^9,10^, which is easily understood and reproducible. The direct measurement was proven as simple and accurate technique for subdural hematoma volume measurement in comparison with volume measure software as this adds extra costs. Lastly, we determined the concrete and critical depressed brain volume as quantified values of 50 cm^3^ according to the ROC curve analysis rather than a simple dichotomy. This process facilitates the prediction of the recurrence in actual practices.

Our study has several limitations. First, our study features a retrospective design and is a single-center study. Second, the follow-up CT scans were evaluated according to our predetermined protocol; thus, the assessment for an optimal follow-up period was an inadequate comparison among depressed brain volumes at pre-operation, postoperative 24 h, and postoperative 7 days instead of a whole perioperative period. Therefore, a large, prospective, multicenter study with long-term follow-up duration is needed to validate our findings.

## Conclusions

Our results indicate that a depressed brain volume >50 cm^3^ shown in CT scans taken at postoperative 7 days is an independent predictor of CSDH recurrence following surgical treatment. Given these findings, these values could be valuable to treat patients with CSDH, as these results can aid in the determination of the appropriate postoperative treatment strategy.
